# Brain-targeted drug delivery - nanovesicles directed to specific brain cells by brain-targeting ligands

**DOI:** 10.1186/s12951-024-02511-7

**Published:** 2024-05-17

**Authors:** Ricardo Moreira, Clévio Nóbrega, Luís Pereira de Almeida, Liliana Mendonça

**Affiliations:** 1 CNC - Center for Neuroscience and Cell Biology, University of Coimbra, Rua Larga, polo 1, Coimbra, FMUC 3004-504 Portugal; 2 CIBB - Center for Innovative Biomedicine and Biotechnology, University of Coimbra, Coimbra, 3004-504 Portugal; 3 Faculty of Pharmacy, University of Coimbra, Coimbra, 3000-548 Portugal; 4 Algarve Biomedical Center Research Institute (ABC-RI), University of Algarve, Faro, 8005-139 Portugal; 5 Faculty of Medicine and Biomedical Sciences, University of Algarve, Faro, 8005-139 Portugal; 6 Institute of Interdisciplinary Research, University of Coimbra, Coimbra, 3030-789 Portugal

**Keywords:** Brain delivery, Nanoparticles, Brain-targeting ligands, Targeting nanoparticles to specific brain cells

## Abstract

Neurodegenerative diseases are characterized by extensive loss of function or death of brain cells, hampering the life quality of patients. Brain-targeted drug delivery is challenging, with a low success rate this far. Therefore, the application of targeting ligands in drug vehicles, such as lipid-based and polymeric nanoparticles, holds the promise to overcome the blood-brain barrier (BBB) and direct therapies to the brain, in addition to protect their cargo from degradation and metabolization. In this review, we discuss the barriers to brain delivery and the different types of brain-targeting ligands currently in use in brain-targeted nanoparticles, such as peptides, proteins, aptamers, small molecules, and antibodies. Moreover, we present a detailed review of the different targeting ligands used to direct nanoparticles to specific brain cells, like neurons (C4-3 aptamer, neurotensin, Tet-1, RVG, and IKRG peptides), astrocytes (Aquaporin-4, D4, and Bradykinin B2 antibodies), oligodendrocytes (NG-2 antibody and the biotinylated DNA aptamer conjugated to a streptavidin core Myaptavin-3064), microglia (CD11b antibody), neural stem cells (QTRFLLH, VPTQSSG, and NFL-TBS.40–63 peptides), and to endothelial cells of the BBB (transferrin and insulin proteins, and choline). Reports demonstrated enhanced brain-targeted delivery with improved transport to the specific cell type targeted with the conjugation of these ligands to nanoparticles. Hence, this strategy allows the implementation of high-precision medicine, with reduced side effects or unwanted therapy clearance from the body. Nevertheless, the accumulation of some of these nanoparticles in peripheral organs has been reported indicating that there are still factors to be improved to achieve higher levels of brain targeting. This review is a collection of studies exploring targeting ligands for the delivery of nanoparticles to the brain and we highlight the advantages and limitations of this type of approach in precision therapies.

## Background

The World Health Organization (WHO) estimates that 1 in every 5 humans suffers from Central Nervous System (CNS) diseases [1]. Neurodegenerative diseases, such as Alzheimer’s disease (AD) or Parkinson’s disease (PD), are becoming more prevalent in today’s increasingly aged societies and are a social and financial burden worldwide [2–4]. Despite the increasing awareness for this problem and the efforts of the scientific community to develop therapeutic strategies, this research field has the poorest success rates in terms of effective drug development [5].

The complex physiology of the human brain, the Blood-Brain Barrier (BBB), and the substantial limitations of most animal models used to study human CNS diseases [6] play an important role in the lack of success in the development of new therapies to treat brain diseases. Considering these hurdles, the rational design of nanoparticles (NPs) prone to be administered in minimally invasive ways (e.g. intravenous administration [IV]) can be a promising approach to overcome some of these limitations [7, 8].

NPs comprise materials with size in the nanoscale in at least one dimension [9, 10]. Such nanomaterials can be part of Nanomedicines that, according to the European Commission’s recommendation, are between 1 and 100 nm in size for at least 50% of the particles [11]. NPs can load a great variety of drugs (small molecules, proteins, nucleic acids, etc.), protecting them from metabolization and elimination from the body, and increasing their half-life in the systemic circulation, raising the probability of drugs to reach their target tissue/organ [7, 8, 12, 13]. The materials to be used in the NPs composition must be, whenever possible, biocompatible and biodegradable in order to reduce immunogenicity and toxicity [14]. Furthermore, NPs’ charge, size, and surface chemistry can be manipulated to improve biodistribution [15, 16]. An important functionalization of NPs is the attachment of hydrophilic polymers to their surface, such as polyethylene glycol (PEG). This hydrophilic polymer creates a “cloud” of water molecules on the surface of the NPs, reducing the opsonization effect and the consequent NPs elimination from bloodstream, increasing their time in blood circulation [17, 18] and their ability to efficiently reach the target cells after IV administration. Additionally, the functionalization of NPs surface by adding targeting ligands makes it possible to direct them to a specific cell type or tissue, increasing the accumulation of the NPs in the tissue/cells and reducing the off-target effects [19, 20].

**Fig. 1:**
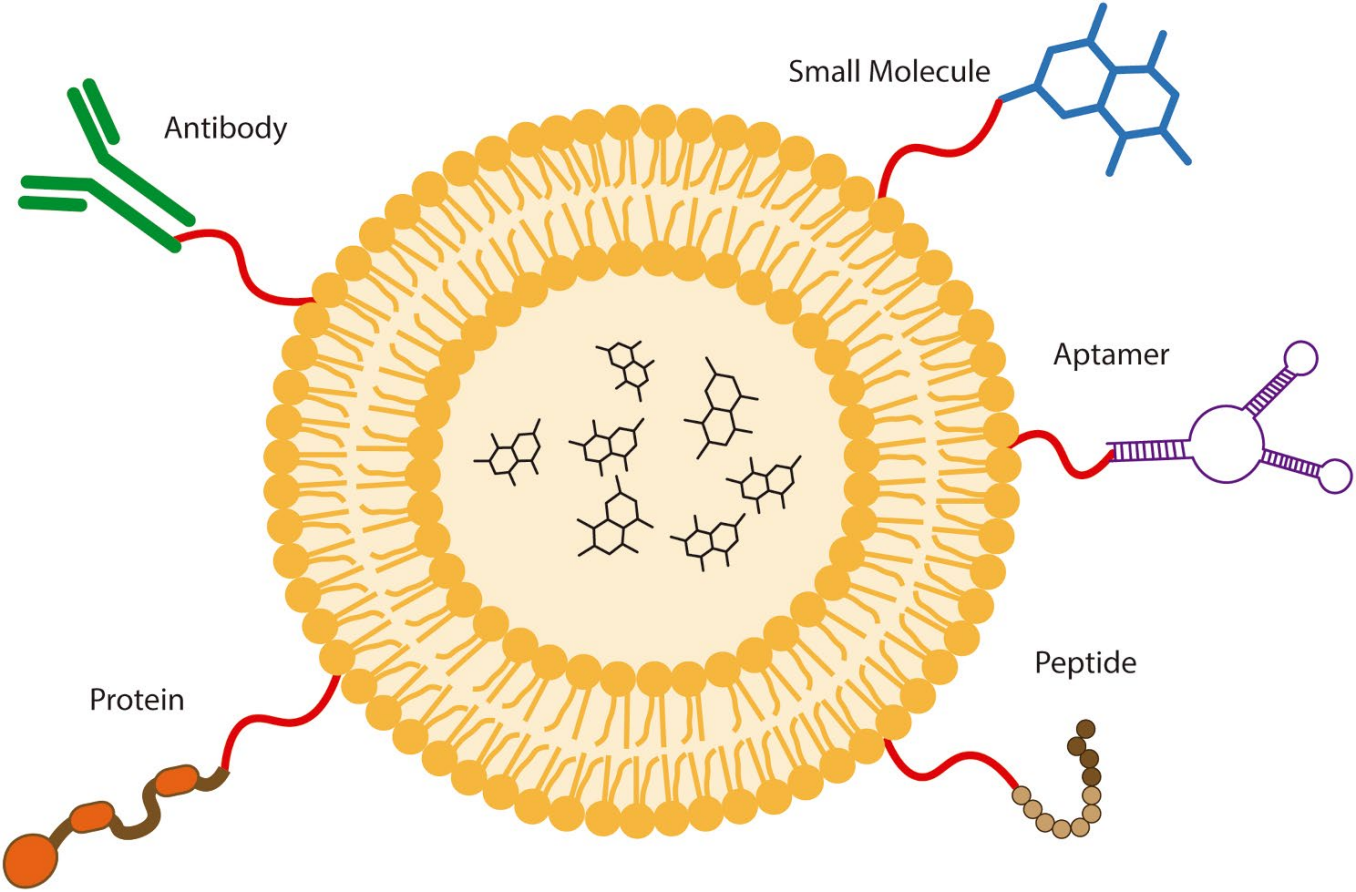
Types of targeting ligands. Several different types of molecules have been employed to achieve specific cellular targeting depending on the characteristics of the NPs used, the goal of the delivery, cost-benefit, and the characteristics of the targeting ligands. Such targeting ligands include antibodies, small molecules, aptamers (RNA/DNA sequences that recognize proteins and receptors with affinity and specificity), proteins, and peptides

The identification of brain-specific ligands that might be employed in the development of brain-targeted NPs is also a critical aspect. Such ligands might specifically direct the NPs to the brain tissue, avoiding unspecific interactions in other compartments, reducing off-target effect and peripheral drug elimination, and consequently enhancing the bioavailability in the brain of the delivered drug. There are different types of targeting ligands that may be employed in the development of NPs (Fig. 1), such as proteins, antibodies, peptides, small molecules, and aptamers, and each of them presents advantages and disadvantages (Table 1) [21–23].

**Table 1:** Different types of targeting ligands available

Types of ligands	Basic structural elements	Advantages	Disadvantages	Clinical use as targeting ligand	Ref.
Antibodies	Aminoacids (high molecular weight)	Strong binding affinity; High specificity	High production cost; Large size; Immunogenicity	Antibody-drug conjugates approved; NPs with antibodies as targeting ligand in Clinical Trials (SGT-94)	[24–29]
Proteins	Aminoacids (high molecular weight)	High specificity	High production cost; Large size	In Clinical Trials (MBP-426, 2B3-101, CALAA-01, 2B3-101)	[30–34]
Peptides	Aminoacids (low molecular weight)	Simple to produce; Small size; High affinity	May be cleaved by proteases in circulation	In Clinical Trials (BT1718, CEND-1)	[35–38]
Aptamers	Synthetic structural RNA/DNA	High specificity; Small size; Customizable for any target	High production cost; May be cleaved by nucleases in circulation	In pre-clinical development (Sgc8, A-10, AS1411, TTA 1)	[39–44]
Small Molecules	Chemical elements (carbon, oxygen, sulfur, etc.)	Low production cost; Small size	Target specificity reduced	In Clinical Trials (SEL-068, BIND-014)	[23, 45–48]

## Blood-brain barrier composition and crossing

**Fig. 2:**
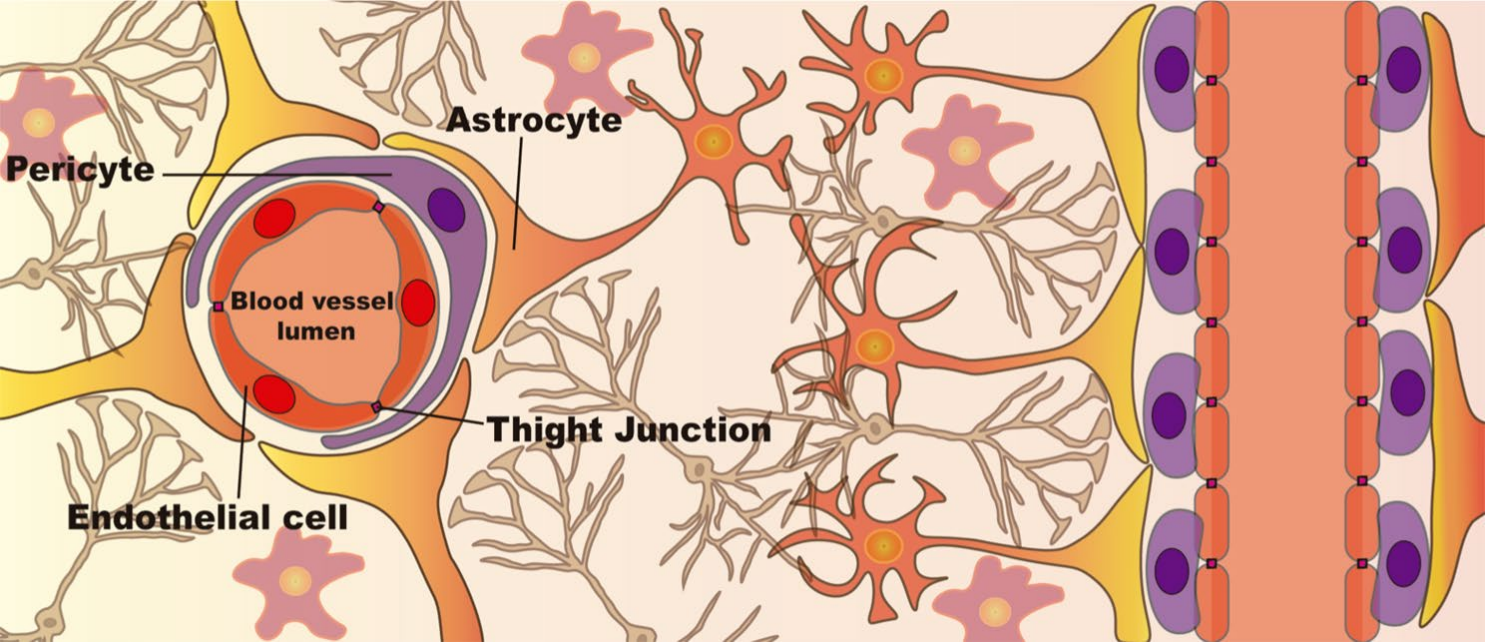
Cellular structure of the Blood-Brain Barrier (BBB). The endothelial cells (red cells) that compose the brain microvasculature are attached to each other by Tight Junctions that bring these cells close together, limiting the passage of unspecific molecules between them. Pericytes (purple cells) are important regulatory cells that involve the endothelial cells. Finally, the endfeet of astrocytes (yellow/orange cells) also involve this structure, providing regulatory support. The BBB strongly suppresses the entry of unwanted pathogens and cells into the brain parenchyma, protecting the resident cells from insults

BBB comprises endothelial cells, pericytes, and astrocytes, building a tight barrier that selectively limits the entry of molecules into the CNS (Fig. 2) [49]. Furthermore, this barrier is characterized by (1) the absence of fenestrations and (2) the presence of tight junctions between endothelial cells and the brain microvasculature formed by claudin, occludin, and junction adhesion molecules [49]. The presence of these molecular tight junctions results in a high transendothelial electrical resistance (1500 Ω/cm^2^ in in vivo measurements [50, 51]), limiting the entry of pathogens and undesired molecules and cells from peripheral circulation into the CNS. However crucial for the maintenance of brain homeostasis, this barrier also hampers the effectiveness of therapies to the brain by limiting their entrance [52, 53]. Less than 1% of the macromolecules and no more than 2% of small molecules are able to cross the BBB by paracellular diffusion [54]. Small hydrophilic and hydrophobic molecules need to have a molecular mass inferior to 150 Da and 400–600 Da, respectively, to be able to cross the BBB by passive diffusion. Consequently, most molecules enter the BBB endothelial cells by endocytosis [55]. After endocytosis, the molecules accumulate in late endosomes, which eventually fuse with lysosomes (forming the phagolysosome), where they can be destroyed by the low pH and hydrolytic enzymes [56]. Thus, the endosomal escape is a key step in the success of therapies that reach the CNS by crossing the BBB [57].

**Fig. 3:**
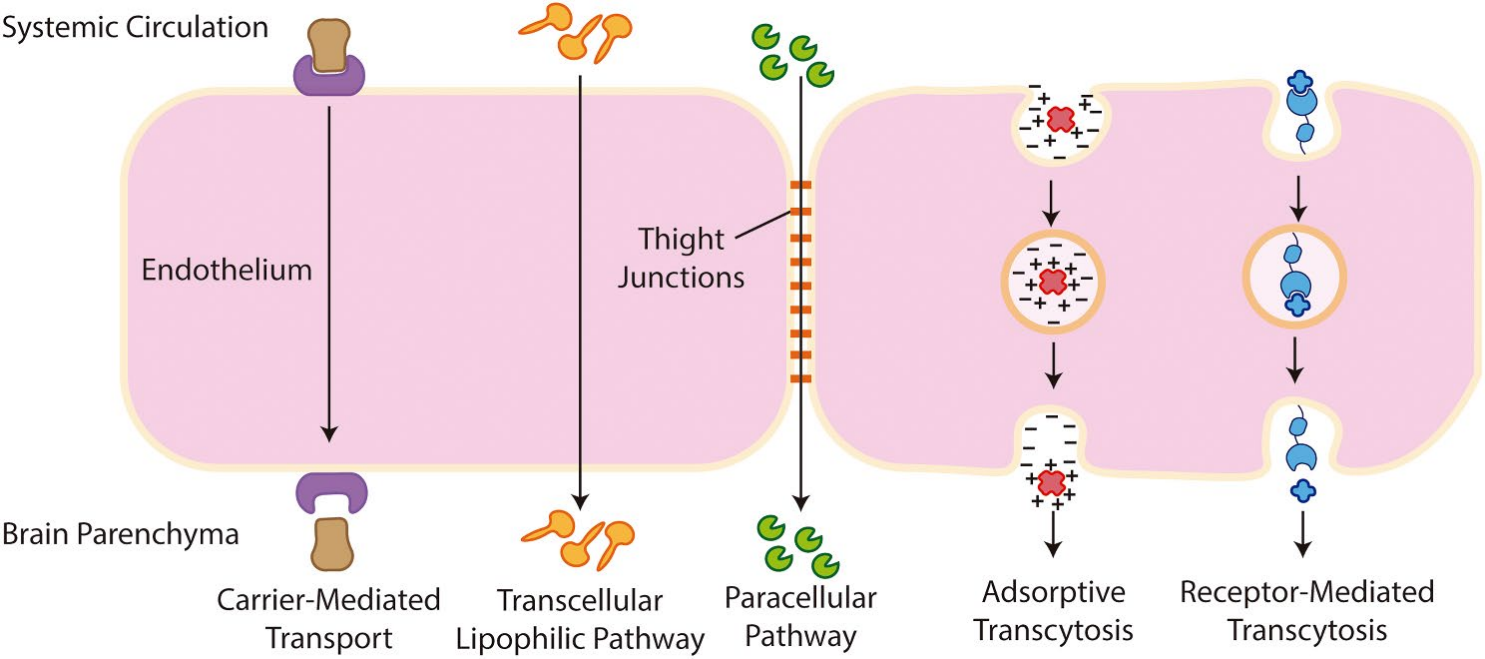
Pathways for molecular transport across the BBB. The cellular and molecular structure of the BBB makes this barrier highly restrictive and selective to molecules that can only cross the BBB through specific mechanisms. Small molecules like glucose are able to enter the brain using for example the glucose transporter Glut-1 as carrier in a Carrier-Mediated Transport. Small lipophilic molecules are able to overcome the BBB via passive diffusion in the Transcellular Lipophilic Pathway. Small hydrophilic molecules, unable to cross through the endothelial cells, are small enough to pass through the Tight Junctions into the brain parenchyma by the Paracellular Pathway. Some cationic molecules are able to interact with the negative charges on the surface of the endothelial cells and cross this barrier in a low capacity and non-specific mechanism called Adsorptive Transcytosis. Finally, large molecules, such as transferrin and insulin, enter the brain parenchyma via specific receptors expressed on the surface of endothelial cells in a mechanism called Receptor-Mediated Transcytosis

Furthermore, the “enzymatic BBB”, which is a complex set of enzymes from brain endothelial cells, promotes chemical compounds degradation [55]. Another key issue regarding the transcytosis of the BBB is the presence of highly efficient efflux pumps in these cells. These efflux pumps, mediated by p-glycoprotein, are responsible for the recognition of molecules that are unnecessary for the brain and transport them back to the vascular lumen, preventing their entry into the brain parenchyma [58]. Accordingly, some studies indicate that the concentration of several drugs is increased in the CNS upon blockage of these efflux transporters [59, 60]. The paracellular aqueous and the transcellular lipophilic pathways allow the passage of very small molecules in between the endothelial cells of the BBB or through them, respectively. Besides these mechanisms, there are other pathways required for large macromolecules to enter the CNS, such as the proteins that enter via receptor-mediated or adsorptive transcytosis (Fig. 3) [61, 62].

In Carrier-Mediated Transport, macromolecules such as glucose, essential fatty acids, and aminoacids, take advantage of transport proteins inserted in the endothelium and use them to transpose the BBB along or against concentration rates. While in receptor-mediated transcytosis, macromolecules such as insulin, epidermal growth factor, LDL, and transferrin bind to specific receptors on the surface of endothelial cells, which activates their endocytosis in the basolateral side of the cells [61, 62]. Finally, in adsorptive transcytosis (non-specific), positively charged ligands interact with the negatively charged cell surface and this interaction promotes endocytosis (Fig. 3).

## Overcoming the blood-brain barrier (BBB)

The most direct way to surpass the BBB is by intraventricular, intrathecal, or intraparenchymal injection of the drugs in the brain or intranasal administration. Several publications demonstrated the successful use of these administration routes when aiming at the delivery of molecular therapies to the brain, which are reviewed elsewhere [63, 64]. However, some of these approaches, namely intraventricular, intraparenchymal, and intrathecal, are highly invasive, requiring very delicate brain surgeries and can cause complications such as spinal cord lesions, seizures, encephalopathy, meningitis, cerebral infection, or subdural empyema [65–67]. In particular, intraventricular injection is associated with a bulk flow of CSF from the ventricles to the subarachnoid space (where major arteries are located), thus causing fast clearance of the injected therapies from the brain [68, 69]. This fast clearance results in the need of frequent dosing, which may impair patient compliance and tolerance to the treatment [63]. The limited drug penetration from CSF to the brain parenchyma, especially for macromolecules is another handicap of this approach [63]. These limitations, and complications related to the devices, namely severe infections, leakage, and immune system activation (presence of white cells in the CSF), have reduced the use of this strategy for brain therapies [63, 70]. As for intraparenchymal administration, the distribution of the therapies in the brain is frequently limited to the site of injection, constraining the therapeutic effect [63, 64], and complications associated with such an invasive surgery have been described [64, 71]. Intrathecal (IT) administration allows access through the perivascular spaces but this approach is highly dependent on the size of the therapy administered [72], and serious adverse effects have been reported related to blood and lymphatic system disorders due to malfunction of port devices for IT which need to be imbedded in the patients for repeated administration [73]. Intranasal administration is a less invasive approach (and more patient-friendly) that allows access to the brain through the nasal epithelium at the level of the cribriform plate, bypassing the BBB, with minimal serum clearance and peripheral metabolism [63, 64]. This promising administration route to deliver therapies into the brain is challenging due to the physicochemical proprieties of the therapies to be delivered that determine their ability to efficiently cross the nasal epithelium and avoid systemic distribution, and the design of the administration device which is crucial to access the specific location in the cribriform plate and allow a controlled administration to both nostrils [63, 64]. A second approach is the use of strategies that transiently promote BBB leakage using compounds to biochemically modulate tight junctions (such as cereport, mannitol, or borneol) or physical methods like hyperosmotic arabinose solutions, electroconvulsive stimulation, laser-induced thermal therapy, or focused ultrasound [5, 74–77]. Nevertheless, this approach carries the risk of brain edema and it also facilitates the invasion of pathogens from the bloodstream [78]. In a third approach, the receptors overexpressed in the BBB have been explored as an entrance gate for the brain, by developing brain-targeting NPs incorporating ligands that target these overexpressed receptors [79], such as the Transferrin receptor (TfR) and the Low-Density Lipoprotein Receptor (LDLR) (Table 2).

**Table 2:** Targeting ligands used for directing therapies for different cell types in the brain

Targeting Ligand	Target Cell Type	Target Receptor	Target Receptor and Tissue specificity	Delivery System	Reference
Transferrin Transferrin antibody (OX26, 8D3, R17217)	Endothelial cells of the BBB	Transferrin receptor	Low tissue specificity; enriched in endothelial cells, bone marrow cells, and monocytes. In the brain is mostly expressed in microvasculature, neurons, and oligodendrocytes.	PLGA Liposomes	[30, 80–84] + HPA*
ApoE Angiopep-2	Endothelial cells of the BBB	Low Density Lipoprotein Receptor (LDLR)	Tissue enriched: adrenal gland; in the brain is mostly expressed in excitatory neurons and endothelial cells of the BBB.	PBCA Human serum albumin-based NPs	[85–89] + HPA*
Insulin Insulin antibody (29B4)	Endothelial cells of the BBB	Insulin receptor	Low tissue specificity; in the brain is mostly expressed in oligodendrocytes.	Human serum albumin-based NPs	[90] + HPA*
Choline	Endothelial cells of the BBB	Choline transporter (SLC5A7)	Tissue enriched: brain and intestine; in the brain is mainly expressed in endothelial cells and some subtypes of neurons.	Dendrigraft poly-L-lysin-based NPs	[91, 92] + HPA*
CRM197	Endothelial cells of the BBB Neurons	Heparin-binding epidermal growth factor-like growth factor (HB-EGF)	Tissue enriched: urinary bladder; increased in endothelial cells	PBCA PLGA	[93] + HPA*
TGN	Endothelial cells of the BBB	N.T.	N.A.	PLA	[94]
VCAM-1 antibody	Endothelial cells of the BBB	VCAM-1	Tissue enriched: lymphoid tissue; increased in vascular endothelium and T-cells; in brain is mostly expressed in microglia.	Lipid nanoparticles	[95] + HPA*
ICAM1 antibody	Endothelial cells of the BBB	ICAM1	Tissue enriched: lung and urinary bladder; in the brain is mainly expressed in microglia.	Lipid nanoparticles	[95] + HPA*
Neurotensin	Neurons	Neurotensin receptor	Tissue enriched: intestine; in brain: 3-fold increased expression in neurons.	Graphene oxide NPs	[96, 97] + HPA*
Tet-1	Neurons	N.T.	N.A.	PEI	[98]
C4-3	Neurons	Tropomyosin receptor kinase B (TrkB)	Tissue enriched: skeletal muscle and tongue; in brain: 3-fold increased expression in neurons.	N.T.	[99] + HPA*
IKRG	Neurons	Tropomyosin receptor kinase B (TrkB)	Tissue enriched: skeletal muscle and tongue; in brain: 3-fold increased expression in neurons.	PCL	[100] + HPA*
Non-toxic carboxylic fragment of the tentanus neurotoxin	Neurons	N.T.	N.A.	Chitosan PEI	[101–103]
Mel kFGF PasR8	Neurons, Astrocytes, Endothelial cells of the BBB	N.A.	N.A.	Liposomes	[104]
pVec QL TAT	Neurons, Astrocytes, Endothelial cells of the BBB	N.A.	N.A.	Liposomes	[105]
Glycoprotein g7	Neurons	Opioid receptor delta 1	Tissue enriched: brain; in brain: 6-fold increased expression in neurons.	PLGA	[106, 107] + HPA*
RVG (RVG-9r, RVG29)	Neurons	nAChR	Tissue enriched: adrenal glands and gastrointestinal tract; in the brain is increased in inhibitory neurons.	Liposomes Human serum albumin-based NPs	[108, 109] + HPA*
AQP4 antibody	Astrocytes	AQP4 channel	Tissue enriched: brain and lung; in brain: specific marker of astrocytes.	PGMA	[110] + HPA*
D4 (GFAP) antibody	Astrocytes	GFAP	Tissue enriched: brain; in brain: specific marker of astrocytes.	Liposomes	[111] + HPA*
Bradykinin B2 antibody	Astrocytes	Bradykinin B2 receptor	Tissue enriched: urinary bladder; increased in excitatory neurons and glial cells.	Chitosan	[112] + HPA*
CD11b antibody	Microglia	CD11b (Integrin subunit α M)	Tissue enriched: bone marrow; increased in brain and immune system tissues; brain marker of microglia and macrophages.	Ceria-zirconia	[113] + HPA*
NG-2 antibody	Oligodendrocyte progenitor cells	NG-2 receptor	Tissue enriched: intestine; increased in the brain, namely in oligodendrocyte progenitors.	PLGA	[114] + HPA*
Myaptavin-3064	Oligodendrocytes	N.T.	N.A.	Streptavidin	[115]
LJM-3064	Oligodendrocytes	N.T.	N.A.	Exosomes	[116]
QTRFLLH VPTQSSG	Neural progenitor cells	N.T.	N.A.	Wild-type adenoviral capsid	[117, 118]
NFL-TBS.40–63	Neural stem cells	N.T.	N.A.	Lipid nanocapsules	[119, 120]
Transferrin	Neural stem cells	N.T.	N.A.	Gold NPs and gold nanorods	[121]

N.A.: not available information; NPs: nanoparticles; N.T.: not tested; PLGA: poly(lactic-*co*-glycolic acid); PLA: poly(lactic acid); PGMA: poly(glycidyl methacrylate); PC: Polycaprolactone; PEI: Polyethylenimine; PBCA: poly(n-butyl cyanoacrylate); nAChR: nicotinic acetylcholine receptor. *data from Human Protein Atlas (https://www.proteinatlas.org/)

The TfR is a glycoprotein widely expressed in several cell types including the BBB endothelial cells, which, although lacks cell-specificity, has been extensively used to target NPs to the brain, especially in cancer [30, 80, 81, 122], given the overexpression of this receptor by cancer cells. Despite the straightforward use of this receptor to target NPs, the high levels of circulating transferrin, which will compete for the TfR, may hamper the targeting of NPs to the BBB. In order to overcome this issue, monoclonal antibodies against TfR, such as OX26, 8D3, and RI7217, were developed to deliver drugs into the brain [82, 83].

Low-Density Lipoprotein Receptor (LDLR) has been tested for both direct- and indirect-brain targeting. Regarding indirect-brain targeting, Kreuter and colleagues observed that coating poly(butyl cyanoacrylate)-NPs, encapsulating loperamide or dalargin (drugs with analgesic properties), with polysorbate 80 enables the adsorption of apolipoprotein E (ApoE) from circulation in their surface, allowing these NPs to target LDLR on the BBB and cross it via receptor-mediated transcytosis [85]. For the direct brain-targeting approach, ApoE was covalently bound to human serum albumin NPs (ApoE-NPs) and IV-injected into SV 129 mice. After 15 and 30 min the animals were sacrificed, their brains removed and evaluated by transmission electron microscopy. Interestingly, only ApoE-NPs were observed inside the brain parenchyma and associated with neurons, while unbound NPs were undetected, demonstrating the targeted delivery of NPs using ApoE [86]. Angiopep-2 is a 19 amino acid peptide that has been shown to target LDLR and to improve brain uptake [87, 88]. Angiopep-2 was conjugated with 3 molecules of the anti-cancer drug paclitaxel and this system tested for breast cancer brain metastasis targeting, since this receptor is overexpressed both in the BBB and brain tumors. The Angiopep-2-conjugated paclitaxel and free drug was tested in mice by IV administration. A 161-fold increase in the brain accumulation and a 12-fold increase in the brain metastasis accumulation of the Angiopep-2-conjugated drug were reported. These results suggest an improved brain and brain metastasis delivery of the drug conjugated with Angiopep-2, compared with free drug [89].

Insulin and monoclonal antibodies targeting the insulin receptor have also been used to direct NPs into the brain. Ulbrich and colleagues prepared human serum albumin NPs covalently bound to insulin or to the anti-insulin receptor monoclonal antibody 29B4 to deliver loperamide (an opiate receptor agonist unable to cross the BBB) into the brain after IV administration in mice [90]. The targeted NPs loaded with loperamide were able to induce significant nociceptive effects in mice evaluated by the tail flick test, as compared with NPs attached to an unspecific IgG. Moreover, a pre-injection of free 29B4 anti-insulin receptor antibody, 30 min prior to insulin-targeted NPs administration, inhibited the antinociceptive effects previously observed with these NPs [90]. Thus, data showed that the use of ligands targeting the insulin receptor enables crossing of the BBB.

The high expression of the choline transporter in the BBB has also been explored for brain targeting. Choline is an essential amino acid and a precursor of the neurotransmitter acetylcholine produced by cholinergic neurons that play an important role in learning and memory [123]. Choline is able to transpose the BBB through the choline transporter present on the surface of brain microvascular endothelial cells [123]. Li and colleagues took advantage of the high expression of Choline transporter in the BBB and glioma cells to achieve a dual targeting with a single ligand [91]. Authors complexed a plasmid encoding for human tumor necrosis factor-related apoptosis-inducing ligand (Trail) and the chemotherapeutic drug doxorubicin (DOX) with dendrigraft poly-L-lysine to establish NPs capable to mediate gene therapy and chemotherapy to tackle glioma. Moreover, a choline derivate ligand, designed with the bis-quaternary ammonium compound isoquinoline that has demonstrated high affinity to the choline transporter in the BBB [92], was used as targeting ligand to overcome the BBB. The higher cellular uptake and therapeutic efficiency of the choline transporter-targeted NPs, compared to the non-targeted NPs, was demonstrated in the U87 MG glioma cell line. U87 MG glioma cells were injected in the right striatum of male Balb/c nude mice, and the choline transporter-targeted and non-targeted NPs were intravenously injected 18 days after the cells’ implantation. NIR images, taken 2 h after NPs administration, demonstrated a preferential accumulation of the choline transporter-targeted NPs in the brain, as compared to non-targeted NPs. However, both types of NPs revealed high accumulation in peripheral organs, especially in the liver and spleen [91].

Heparin-binding epidermal growth factor-like growth factor (HB-EGF) is another membrane bound receptor widely expressed in the cerebral blood vessel endothelia, neurons, and glial cells [124]. It has been demonstrated that the carrier protein CRM197 is able to mediate the BBB-targeted delivery using receptor-mediated endocytosis via HB-EGF [125]. CRM197 is a mutated form of the diphtheria toxin produced by the bacteria *Corynebaterium diphtheriae* that when released in the bloodstream may cause neuritis [126]. CRM197 targeting ligand has been used with success [93, 127]. For example, using an in vitro BBB model composed of human brain-microvascular endothelial cells (HBMEC) seeded on the top (Polyester membrane) of a transwell and human astrocytes seeded on the bottom, Kuo and colleagues investigated the ability of polybutylcyanoacrylate (PBCA) NPs conjugated with CRM197 to deliver zidovudine (AZT). The NPs were loaded with dextran-FITC and their uptake in HBMEC was demonstrated by fluorescent microscopy [93]. Similarly, the ability of CRM197 to deliver polymeric poly-lactide (PLGA) NPs to the brain of CD1 wild-type mice after IV administration was assessed [127]. CRM197-targeted NPs loaded with the rhodamine B dye were administered to the mice, which were sacrificed 30 and 60 min after the administration. For both time points, red spots were observed in whole brain parenchyma, indicating the presence of the NPs. It was also reported significant accumulation of the CRM197-NPs in the liver and spleen and limited uptake in the kidneys and lungs. The cellular tropism of the CRM197-NPs was evaluated 30 min, 6 and 48 h after administration. A preferential accumulation in NeuN-positive cells (neurons) was detected. Additionally, over time there was an increased accumulation of these NPs, being reported that 40%, 48%, and 63% of the cells co-localized with the NPs for each time point, respectively. GFAP-positive cells (astrocytes) presented 35% of co-localization with NPs at 30 min, but their presence was decreased to 15% and 2% for 6 and 48 h, respectively. Furthermore, CRM197-NPs loaded with loperamide were intravenously injected in mice to test their ability to trigger nociceptive effects. Five hours post administration, the analgesic effect reached 35% and remained high for 2 days. Whereas, the control groups, namely free loperamide and unloaded CRM197-NPs, were unable to trigger analgesic effect. The untargeted loperamide-loaded NPs showed reduced analgesic activity with maximum possible effect (MPE) values between 5 and 10% [127].

**Fig. 4:**
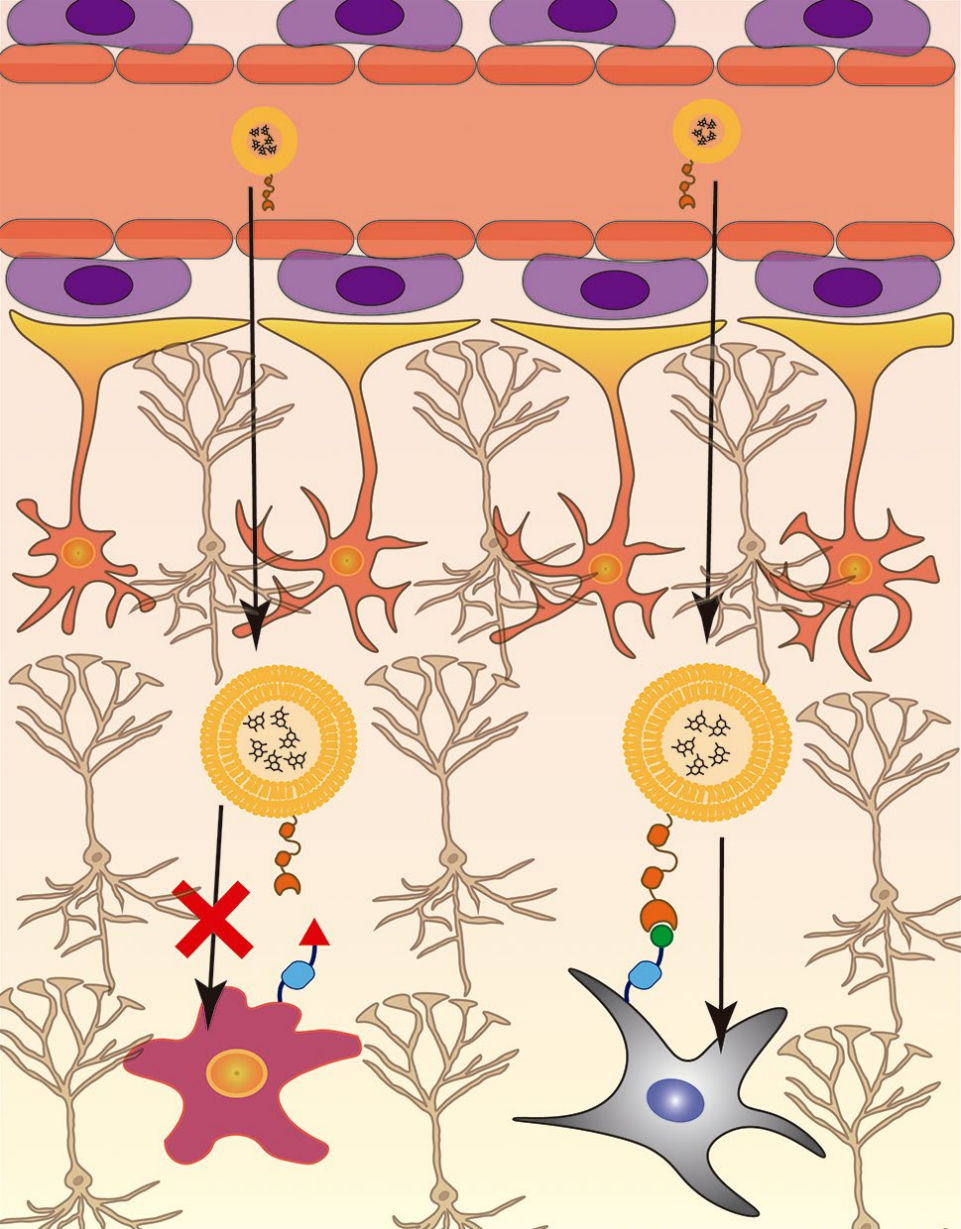
Cell specific targeting. The presence of specific receptors or overexpression of certain receptors on the cell surface may be explored to promote a targeted delivery of the NPs to such cells. NPs formulated with a specific targeting ligand are unable to enter cells lacking the specific receptor for the targeting ligand as illustrated by the purple cell. On the other hand, the NPs are able to specifically deliver its cargo to the cells expressing the receptor specific for the targeting ligand, illustrated by the gray cell

Brain inflammation is a critical condition observed in most neurodegenerative diseases [128–130], promotes significant alterations in the BBB, including enhanced leakage of this structure, further increasing neuroinflammation and brain edema [95, 131, 132]. Some studies explored this inflammatory status to target therapies to the brain, namely by targeting specific markers of inflammation in the endothelium. In particular, Marcos-Conteras and colleagues developed NPs loaded with mRNA of thrombomodulin (a factor produced by endothelial cells that is responsible for inhibiting thrombosis, vascular leakage, and inflammation) using as targeting ligand an antibody to vascular adhesion molecule 1 (anti-VCAM-1) and compared their delivery capacity to TfR- and anti-intracellular adhesion molecule 1 (anti-ICAM1)-targeted liposomes [95]. ICAM1 is expressed in endothelial cells, including vascular endothelial cells, as a surface receptor and its expression is described to be enhanced in pathological conditions [133]. Regarding VCAM-1, this receptor is specifically expressed on the surface of vascular endothelial cells and was described as overexpressed in neuroinflammation, serving as one of the initial players to this process [134]. The delivery capacity of the NPs was tested in C57Bl/6 mice with acute brain inflammation induced by microinjection of TNFα in the striatum. The brain accumulation of liposomes using anti-VCAM-1 as targeting ligand was 27- and 8-fold enhanced compared to liposomes with anti-TfR and anti-ICAM1, respectively. Additionally, lipid NPs conjugated with anti-VCAM-1 and loaded with mRNA of thrombomodulin selectively accumulated in the inflamed brain and the *de novo* expression of the cargo mRNA resulted in alleviation of TNFα-induced brain edema [95]. Additionally, to improve the targeted delivery, after overcoming the BBB it is important to direct NPs to specific cells in the brain parenchyma. In this regard, several strategies exploring the specific recognition by targeting ligands of the different resident cells in the brain, namely neurons, astrocytes, microglia, oligodendrocytes, and neural stem cells, (Fig. 4) have been developed and will be discussed in the following sections.

### Targeting brain tumors

The most common primary malignancy in the CNS is glioma, which, due to its infiltrative growth and difficulty to be removed surgically, is associated with poor prognosis and short survival rates [135, 136]. In this regard, extensive work has been done aiming at the development of anti-cancer medicines capable to overcome the BBB and target glioma using NPs as drug carriers [137–140]. Interestingly, TfR and LDLR are described to be overexpressed in glioma cells and in endothelial cells of the BBB, marking them attractive targets in the development of such therapies [141–145]. Beside the challenge to overcome the BBB, glioma therapy also faces the hurdle to penetrate the tumor. As so, Zhu and colleagues developed docetaxel-loaded nanomicelles coupled with two targeting ligands, Angiopep-2 and TAT [146]. As discussed above, Angiopep-2 is a peptide that targets LDLR, while TAT is a cell penetrating peptide (CPP). TAT was linked to a short PEG_2000_, shielded by a longer PEG_6000_ to avoid unspecific cell penetration during circulation in the bloodstream. Authors argue that after coupling of Angiopep-2 to its target receptor, the close contact between NPs and endothelial cells triggers the effect of TAT, enhancing the crossing of the BBB and further accumulation in the glioma [146]. Several different ratios of the two ligands in the NPs were tested and the combination of 20 mol% of Angiopep-2 with 10 mol% of TAT resulted in higher cell uptake of the NPs compared to single targeted Angiopep-2 micelles and non-targeted micelles. To study the pharmacokinetics of the NPs, authors labeled the docetaxel-loaded micelles with Cy-5 and injected them into Balb/C mice. Comparing to free drug, all micelles (non-, double- or single-targeted) presented over 10-fold higher circulation times. Moreover, the double-targeted NPs exhibited more pronounced drug delivery to the brain. Importantly, the accumulation observed in peripheral organs for double-targeted NPs was relatively low, indicating that the shielding of TAT with PEG was successful. Regarding antitumor efficacy of the double-targeted NPs, the formulation was injected in Balb/C nude mice bearing an orthotopic U87MG glioma. The docetaxel loaded double-targeted NPs were more efficient in inhibiting tumor growth, resulting in pronounced reduction of body weight loss, and increase in survival time up to 2-fold, with residual damage of peripheral organs [146].

Zhu and colleagues also established a formulation based in reduction-sensitive Polycaprolactone (PCL) micelles, functionalized with cyclic RGD peptide, to deliver DOX to U87MG glioma xenografts [147]. cRGD has high affinity for α_v_ β_3_ integrins, which are described to be highly expressed on malignant tumor cells like U87MG [148, 149]. Beside the lack of a targeted approach, the slow drug release from their vehicle also causes poor efficacy of antitumor therapy [150–152]. Hence, the authors took advantage of the reductive environment in cancer cells [153, 154], to develop micelles with a S-S (disulfide) linker between PCL and PEG in order to enhance the NPs destabilization once inside the cancer cells and consequently promote DOX release. DOX release in U87MG cells was 2.3- and 4-fold increased for cRGD/PEG-SS-PCL micelles compared to non-targeted PEG-SS-PCL and reduction insensitive cRGD/PEG-PCL micelles, respectively [147]. In nude mice xenotransplanted with U87MG cells, cRGD/PEG-SS-PCL and cRGD/PEG-PCL micelles exhibited 2.2-fold increase accumulation in the tumor site compared to non-targeted PEG-SS-PCL micelles (4.38% ID/g and 4.12% ID/g VS 1.99% ID/g, respectively), with lower DOX accumulation in liver and heart. Moreover, the DOX signal at the tumor site for cRGD/PEG-PCL micelles was weaker than the signal for cRGD/PEG-SS-PCL, indicating an enhanced DOX release promoted by the latter micelles. Regarding tumor growth, cRGD/PEG-SS-PCL significantly inhibited tumor growth by 50% compared to cRGD/PEG-PCL and PEG-SS-PCL micelles [147], demonstrating the therapeutic efficiency of DOX delivered by the cRGD/PEG-SS-PCL micelles.

The dysregulation of gene expression in glioblastoma cells, namely of microRNAs like miR-21, has been associated with tumor development and progression [155]. As so, modulation of these miRNAs with oligonucleotides (ODNs) has been demonstrated to reduce migration and proliferation of glioblastoma cells and increase the cytotoxic effect of anticancer drugs [156, 157]. With this in mind, Costa and colleagues developed stable nucleic acid lipid particles (SNALPs) loaded with anti-miR-21 ODNs and using chlorotoxin (CTX) as targeting ligand [158]. CTX is reported to bind to matrix metalloproteinase 2 (MMP-2), which is considerably overexpressed in glioblastoma compared to normal tissues [159]. Using FAM-labeled anti-miR-21 ODNs in CTX-targeted and non-targeted SNALPs, the authors observed an almost 10-fold increase in fluorescence signal for CTX-SNALPs compared to non-targeted NPs, indicating that CTX significantly increases the internalization of SNALPs by U87MG cells. Furthermore, CTX-SNALPs promoted a 5-fold reduction in miR-21 expression in these cells compared to non-targeted SNALPs, which had no effect on miR-21 expression. Interestingly, miR-21 silencing resulted in increased expression of PTEN and PDCD4, two tumor suppressors modulated by miR-21 [160, 161]. Moreover, a reduction in the antiapoptotic effect, by a 2-fold increase in caspase 3/7 activity, was also observed. For in vivo experiments, CTX- and non-targeted SNALPs were administered into a glioblastoma mouse model, established through GL261 cell (mouse glioblastoma cell line) injection in the mice brain. A 2-fold accumulation of CTX-SNALPs compared to non-targeted particles was observed in the transplanted glioblastoma cells [158].

Up to 20% of cancer patients will develop brain metastases, leading to poor prognosis and reduced survival rates with current state-of-the-art treatments [162–164]. Pharmacological access to these brain metastases is a major hurdle, with reported drug concentrations 10 times lower in brain metastases compared to other metastases, which is explained in part by the presence of the BBB [165, 166]. Prostate-specific membrane antigen (PSMA) is a receptor described to be overexpressed in BBB endothelial cells of newly formed vasculature feeding the brain metastases, while PSMA detection on regular endothelial cells of the BBB is residual [167, 168]. Taking advantage of this different PSMA expression, Ni and colleagues developed PLGA-NPs employing a double-targeting system approach. Thus, NPs were conjugated to the small molecule ACUPA, which has been described as an efficient targeting ligand for PSMA [169, 170], to target the brain metastases endothelial vasculature, and the peptide cyclic TT1 (cTT1) which has demonstrated tumor-targeting abilities [162, 171]. The in vivo evaluation of the NPs was performed in mice bearing breast cancer cell metastases (BCBM), induced by intracardiac injection of 231Br cells (human breast cancer cell line). The NPs were loaded with DOX or Lapatinib (LAP); both types of NPs were co-injected to achieve synergistic activity between both drugs. After injection in BCBM mice, ACUPA (A)-NPs and A-NPs-cTT1 enhanced brain accumulation, while no significant accumulation was observed in peripheral organs. Moreover, treatment with DOX and LAP loaded A-NPs-cTT1 led to tumor growth reduction compared to free drug and non-targeted NPs. Finally, animals treated with A-NPs-cTT1 had an extended median survival time (44 days) compared to saline (25 days), free combination (29 days), non-targeted NPs (29 days), A-NPs (33 days), and NPs-cTT1 (32 days) [162].

### Targeting neurons

Neurons are specialized brain cells responsible to process and transmit information to other cells via electrical and chemical signals [172]. Therapies that specifically target these cells are particularly important since they are the major cell type affected in neurodegenerative diseases [173, 174]. Typically, neurodegenerative diseases affect one specific subset of neurons, leading to the dysfunction of specific brain regions [174]. For example, neurons from the hippocampus and the cerebral cortex, which mostly express M1 and M2 muscarinic acetylcholine receptors, are the most affected in AD [175], while neurons from the striatum, which in turn express more M4 muscarinic acetylcholine receptors, are more affected in PD [176]. Given these differences between neurons of different brain regions, it is important to select an appropriate ligand that is able to target the specific cells in the brain aimed to be treated [20]. Although challenging, some work has been done in order to develop NPs that specifically target neurons in the context of several neurodegenerative diseases [20, 177–179].

Neurotensin neuropeptide has been demonstrated to be specifically internalized by neurons via receptor-mediated uptake [96]. To target neurons, Hsieh and colleagues coupled Neurotensin to graphene oxide NPs, functionalized with polyethyleneimine (PEI) in order to obtain positively charged NPs [97]. Taking advantage of external destabilization of the cellular membrane using near-infrared (NIR) laser irradiation, the mentioned NPs were used for plasmid DNA (pDNA) delivery specifically into neurons. In vitro, the described system was able to deliver pDNA in PC-12 cells differentiated into neuron-like cells. Upon intracerebral injection in the caudate nucleus of C57Bl/6 mice, the NPs not coupled to neurotensin transfected mostly glial cells. Whereas, neurotensin-coupled NPs transfected mostly neurons [97].

Park and colleagues compared PEGylated neurotensin-coated PEI NPs (NT-PEI) with Tet-1-coated NPs [98]. Tet-1 is a peptide with the binding characteristics of the tetanus toxin, which interacts specifically with motor neurons and has the ability to undertake retrograde transport to the cell soma [98]. The NPs (NT-PEI, Tet1-PEI, and PEI (control)) labeled with the YOYO-1 fluorophore were added to neuron-like differentiated PC-12 cells. Flow cytometry analysis revealed that the PEI-treated cells had a similar fluorescence profile as untreated cells (0.6% of cells). While cells treated with the targeted NT-PEI and Tet1-PEI NPs presented 12.7% and 16.3% higher fluorescence levels, respectively. Furthermore, as the Tet1-PEI NPs revealed higher binding affinity to neuron-like cells, it was also demonstrated, through confocal microscopy, that neuronal cultures internalize the Tet1-PEI NPs [98].

The Tropomyosin receptor kinase B (TrkB) is a receptor abundantly expressed by neurons, being activated by BDNF and internalized upon activation. This receptor is key to neuronal survival, plasticity, and neuroregeneration [180]. Therefore, it might be an interesting entrance gate in neurons. Accordingly, Huang and associates developed a screening platform for aptamers that target this receptor [99]. The C4-3 aptamer was identified as an agonist for TrkB and was tested in primary cultures of embryonic rat cortical neurons. Data revealed an increase in phosphorylated TrkB (p-TrkB) (the activated form of this receptor), as well as increased neuroprotection when the cells were deprived of supplements in their culture media [99]. To test the agonist activity of C4-3 in vivo, this aptamer or a scrambled (control) aptamer were injected into the hippocampus of adult mice. Increased p-TrkB levels were observed in the hippocampus of C4-3-injected mice, which was not detected in mice injected with the scrambled aptamer, demonstrating the agonist activity of C4-3 in vivo [99]. In line with this work, Xu and colleagues developed IKRG, a tetra peptide that mimics BDNF function and interacts with TrkB promoting its internalization, to be used as a targeting ligand for neurons in polymeric polycaprolactone (PCL) NPs functionalized with PEG [100]. In a proof-of-concept study, the authors started to evaluate the uptake of PEG-PCL NPs functionalized with IKRG to selectively target TrkB. The ability of these NPs to be internalized by TrkB-expressing (PC-12) and non-expressing (HeLa) cells was tested. Data indicated that IKRG-NPs were only internalized by TrkB-expressing cells. Furthermore, the authors evaluated the ability of these NPs to deliver VO-OHpic, an inhibitor of PTEN (Phosphatase and tension homolog deleted on chromosome 10), in order to promote neuroregeneration in peripheral neuropathies. For this, the NPs were tested in primary cell cultures obtained from the dorsal root ganglion of C57Bl/6 mice, composed of neurons, Schwann cells, fibroblasts, and glial cells. Successful and preferential internalization of the IKRG-NPs in neurons was reported, as demonstrated by the 2-fold increase in the co-labeling of NPs with TUJ-1 (a neuron-specific marker), compared to untargeted NPs [100].

Lopes and colleagues tested a non-toxic carboxylic fragment of the tetanus neurotoxin heavy chain with 54 kDa and neurotropic properties, which is able to undergo active retrograde transport after peripheral administration [101–103]. In this work, the authors took advantage of the neuron-targeting properties of this fragment to direct polymeric NPs composed by thiolated trimethyl chitosan, loaded with pDNA encoding for BDNF. These NPs were tested in a mouse model of peripheral nerve injury, in order to restore enervation and neuroregeneration after intramuscular administration [103]. This delivery system promoted a significant expression of BDNF in neurons, compared to vehicle or non-targeted NPs, followed by neuroregeneration and functional recovery after injury. Additionally, data revealed an increase in the expression of neurofilament heavy chain (associated with neuroregeneration) and GAP-43 (a protein associated with axonal growth) proteins in the site of injury, a significantly higher density of myelinated axons, increased pAKT expression, and enhanced neurite outgrowth and density [103], demonstrating the targeted delivery potential of this fragment of the tetanus neurotoxin.

Cell-penetrating peptides (CPP) are small, relatively non-toxic peptides (with less than 30 aminoacids) that were discovered 30 years ago and since then have been used to deliver different kinds of cargo to cells, including pDNA, small interfering RNA (siRNA), viruses, small molecules, and even therapeutic proteins and NPs [181]. These peptides can be derived from natural proteins (such as viral and antimicrobial proteins), chimeric, or completely synthetic [181]. The exact mechanism of how CPP are able to enter the cell is still a matter of debate. Endocytosis and direct penetration of the cell membrane are the two more likely cell entry mechanisms for CPP and are highly dependent on the type of CPP, its concentration, cargo, and the cell type [181]. For example, one of the first CPP to demonstrate the ability to enter differentiated neurons was a DNA-binding peptide, a 60 aminoacids region of the antennapedia homeobox protein (pAntp) from *Drosophila* [182]. Moreover, Santos Rodrigues and co-workers tested the ability of liposomes functionalized with transferrin and CPP to accumulate in different cell types (endothelial cells, astrocytes, and neurons) [104]. In this experiment, three different CPPs, Mellitin (Mel), Kaposi fibroblast growth factor (kFGF), and a conjugation of the penetration accelerating sequence (Pas) with the arginine-rich peptide R8 (PasR8), were tested together or not with transferrin. Mel is a 26 aminoacids cationic peptide derived from bee venom, which causes the rearrangement of the cell’s plasma membrane to form pores upon contact, facilitating the entry of cargo into the cell [183, 184]. kFGF is a hydrophobic peptide with the ability to non-covalently bind to DNA by complexation, protecting the cargo from nucleases, and successfully delivering it to cells [185, 186]. Pas is also a hydrophobic peptide (FFLIPKG) that when added to the arginine-rich R8 peptide forms a hybrid peptide with enhanced carrier abilities and capacity to evade lysosomes [187, 188]. The ability of the functionalized liposomes to efficiently deliver pDNA encoding for green fluorescent protein (GFP) to neurons isolated from newborn rats was evaluated 48 h after incubation. Interestingly, liposomes with dual functionalization (conjugated with two ligands) transfected more cells than single-functionalized liposomes conjugated with one of the CPPs. Neurons displayed 5% of transfection after incubation with non-functionalized liposomes vs. 7%, 18%, 8%, 20%, 6%, and 10% of transfection with liposomes functionalized with Mel, Mel + Tf, kFGF, kFGF + Tf, PasR8, and PasR8 + Tf, respectively. Furthermore, liposomes were loaded with lissamine rhodamine, administered in the tail vein of C56Bl/6 mice, and biodistribution was evaluated through the relative fluorescence intensity measured using near-infrared (NIR) imaging. The fluorescence in the brain of the mice injected with the liposomes functionalized with kFGF + Tf was increased. The latter NPs resulted in higher brain accumulation (5.7% of the injected dose/g [ID/g]), as compared with 2.3%, 2.7%, 3.2%, 2.1%, and 3.7% of brain accumulation obtained for liposomes conjugated with kFGF, Mel, Mel + Tf, PasR8, and PasR8 + Tf, respectively. Despite these encouraging results, a significant accumulation of the NPs in the liver (14.6% ID/g), kidneys and lungs (4.8–10.4%), hearth (5.4%), and spleen (3.2%) was also reported [104]. These authors also tested the conjugation of liposomes with Tf and other CPPs, such as the vascular endothelial-cadherin-derived peptide (pVec), the pentapeptide QLPVM (QL), and the HIV-1 trans-activating protein (TAT) [105]. pVec is an 18 aminoacids amphipathic peptide, which presents a hydrophilic end that interacts with the cell membrane and another hydrophobic end that destabilizes the membrane allowing the entry of the CPP into the cell [189, 190]. QL is a hydrophobic pentapeptide derived from the Bax-binding domain of the Ku-70 protein that has cell permeability ability [191, 192]. TAT is a cationic peptide that was the first CPP to be characterized [193]. TAT owes its cell-penetrating capacity to its positive charges that interact with the negative charges of glycosaminoglycans present at the cell surface [194]. Using the same methodology, it was evaluated the transfection ability of single-functionalized (with one of the 3 CCPs) and dual-functionalized (Tf and one of the 3 CPPs) liposomes loaded with pDNA encoding for GFP. As previously observed, the dual-functionalized liposomes outperformed their single-functionalized counterparts. Moreover, TAT (single and dual) functionalized liposomes demonstrated the best delivery capacity. The number of transfected neurons was 4% for non-functionalized liposomes compared with 7%, 10%, 6%, 8%, 9%, and 13% for liposomes functionalized with pVec, pVec + Tf, QL, QL + Tf, TAT, and TAT + Tf, respectively [105]. In vivo, a brain accumulation of 7.7% for TAT + Tf-liposomes and 3.1% for TAT-liposomes was reported. Additionally, the authors also reported a considerable accumulation of these liposomes in the liver and kidneys, and TAT-liposomes were also found accumulated in the lungs [105]. Despite interesting, the poor tissue specificity observed when applying CPPs in delivery systems [195] raises concerns regarding accumulation in off-target cells.

The glycopeptide 7 (g7) has brain-targeting ability. This peptide was engineered from the opioid peptide MMP-2200 through the replacement of the aminoacid Tyr (responsible for the opioid effect) by Phe [107, 196]. Thus, g7 peptide conjugated in PLGA NPs was tested to overcome the BBB and accumulate in neurons [106]. These NPs were injected, via intraperitoneal (i.p.) administration, in C57Bl/6 mice and a brain accumulation of up to 10% of the injected dose was reported. Furthermore, neurons were the main cell type targeted by the NPs, although affinity to microglia and minor co-localization of the NPs with astrocytes was also detected. Interestingly, region-specific brain accumulation of the NPs was reported, namely into some subtypes of neurons, such as neuropeptide Y (NPY) and glutamic acid decarboxylase (GAD) positive interneurons. Moreover, interaction studies revealed a clathrin-dependent internalization mechanism in the NPs’ internalization by the neurons [106].

The rabies virus glycoprotein (RVG) is the glycoprotein responsible for the neurotrophic nature of the rabies virus [197]. The receptor of the nervous system responsible for the interaction with RVG is still a matter of debate; nevertheless, the nicotinic acetylcholine receptor (nAChR) was the first receptor identified to play a key role in this interaction [198]. Derivates of the RVG have been explored to target NPs to the brain. These peptides are shorter versions of the original RVG, which retain the capacity to target and be internalized by neurons. For example, in the context of Machado-Joseph disease (MJD), a neurodegenerative disease presenting extensive neuronal death caused by the mutant ataxin-3 presence in neurons, our group developed RVG-9r-conjugated liposomes encapsulating siRNA to silence mutant ataxin-3 [108]. RVG-9r, a ligand derived from RVG with 9 arginine residues, was used as a brain-targeting ligand that enables the BBB transpose. The biodistribution data of RVG-9r-liposomes (encapsulating the near-infrared dye (NIR) indocyanine green (ICG)) IV injected in mice showed that the RVG-9r targeting ligand increased by 20% the brain accumulation of liposomes, compared to a control ligand. The RVG-9r targeting ligand also led to 25% and 30% decrease in liposomes accumulation in the heart and lungs, respectively, compared to the control ligand. Furthermore, the administration of RVG-9r-liposomes encapsulating siRNA for mutant ataxin-3 silencing, in an MJD transgenic mouse model [199], resulted in 30% reduction in mutant *ATXN3* mRNA, as compared with RVG-9r-liposomes encapsulating a control siRNA [108]. These data indicate that RVG-9r-mediated delivery of liposomes encapsulating gene silencing therapies is an efficient approach to silence mutant ataxin-3 in MJD. Moreover, a peptide derived from RVG with 29 amino-acids (RVG-29) was used by Chen and colleagues to target human serum albumin NPs loaded with the antifungal drug itraconazole (ITZ) to treat brain fungal infections [109]. NPs conjugated or not with RVG-29 were injected into the caudal vein of adult mice. Significantly increased levels of ITZ in the group of animals injected with RVG29-conjugated NPs, as compared to control/untargeted liposomes, was reported. Namely, 2 h post-injection, 100 ng of ITZ/g of brain tissue was detected for the untargeted NPs. Whereas, 200 ng of ITZ/g of brain tissue was detected for the RVG-29-conjugated NPs. These data showed that RVG-29-conjugated NPs could be exploited as a brain delivery system [109].

Despite the encouraging data reported for brain-targeted NPs, these reports also highlight the need to develop more specific brain-targeting ligands and/or NPs to avoid their accumulation in peripheral organs, which results in loss of NPs in undesired sites and also to potential off-target effects (Table 3).

**Table 3:** Studies comparing the accumulation of targeted NPs in the brain with peripheral organs

Formulation	Brain accumulation*^1^	Peripheral organs accumulation*^1^	Reference
Radiolabeled Tramadol (with ^99m^Tc)-loaded PLGA nanoparticles with transferrin as targeting ligand	0.24% ID/g	Liver: 20% ID/g Spleen: 21% ID/g Heart: 1.3% ID/g Kidneys: 10% ID/g Lungs: 3% ID/g	[80]
Lissamine rhodamine-loaded and Mellitin-conjugated liposomes	2.7% ID/g	Liver: 16% ID/g Kidneys: 8.5% ID/g Lungs: 10% ID/g Heart: 4% ID/g Spleen: 2% ID/g Blood: 2.5% ID/mL	[104]
Lissamine rhodamine-loaded liposomes with Mellitin and transferrin as targeting ligands	3.2% ID/g	Liver: 17% ID/g Kidneys: 5% ID/g Lungs: 5% ID/g Heart: 2% ID/g Spleen: 1% ID/g Blood: 1% ID/mL	
Lissamine rhodamine-loaded and kFGF-conjugated liposomes	2.3% ID/g	Liver: 14% ID/g Kidneys: 5% ID/g Lungs: 4.5% ID/g Heart: 3.5% ID/g Spleen: 1.5% ID/g Blood: 2.5% ID/mL	
Lissamine rhodamine-loaded liposomes with kFGF and transferrin as targeting ligands	5.7% ID/g	Liver: 14% ID/g Kidneys: 10% ID/g Lungs: 10% ID/g Heart: 6% ID/g Spleen: 3.5% ID/g Blood: 3.5% ID/mL	
Lissamine rhodamine-loaded and PasR8-conjugated liposomes	2.1% ID/g	Liver: 14% ID/g Kidneys: 8% ID/g Lungs: 8% ID/g Heart: 2% ID/g Spleen: 1.5% ID/g Blood: 3.5% ID/mL	
Lissamine rhodamine-loaded liposomes with PasR8 and transferrin as targeting ligands	3.7% ID/g	Liver: 14.5% ID/g Kidneys: 8.5% ID/g Lungs: 10% ID/g Heart: 2% ID/g Spleen: 1% ID/g Blood: 2.5% ID/mL	
Lissamine rhodamine-loaded and TAT-conjugated liposomes	3.1% ID/g	Liver: 6% ID/g Kidneys: 7.5% ID/g Lungs: 9% ID/g Heart: 3.5% ID/g Spleen: 3% ID/g Blood: 3% ID/mL	[105]
Lissamine rhodamine-loaded liposomes with TAT and transferrin as targeting ligands	7.7% ID/g	Liver: 10% ID/g Kidneys: 9% ID/g Lungs: 3.5% ID/g Heart: 4% ID/g Spleen: 1% ID/g Blood: 3% ID/mL	
Rhodamine 123-loaded and g7-conjugated PLGA nanoparticles	15.89% ID/g	Liver: 17.5% ID/g Spleen: 7.66% ID/g Lung: 13.78% ID/g Kidneys: 26.87% ID/g	[107]
ICG-loaded and RVG-9r-conjugated liposomes	+ 17%*^2^	Heart: -23%*^2^ Lungs: -30%*^2^ Liver: +5%*^2^ Spleen: +1%*^2^ Kidneys: +105%*^2^	[108]

*^1^highest detected concentration for each organ

*^2^fold-quantifications comparing to liposomes with a random peptide on the surface using NIR signal

PLGA: poly(lactic-*co*-glycolic acid); ICG: indocyanine green; %ID/g: percentage of injected dose per gram of animal

### Targeting astrocytes

Astrocytes have key functions in neurotrophic, physical, and metabolic maintenance to neurons, and are indispensable in neurotransmission, namely in supporting and modulating synapses [200–204]. Additionally, astrocytes contribute to immune surveillance in the brain becoming activated in insults, infections, and brain diseases, releasing inflammatory mediators [205, 206]. Several neurodegenerative diseases, such as AD, PD, Huntington’s disease (HD), and Amyotrophic Lateral Sclerosis (ALS), affect astrocytes (reviewed in [207]), requiring their treatment and consequently drug targeting. The most employed delivery system targeted to astrocytes are viral vectors, since virus can be engineered to have pseudo-tropism for astrocytes and astrocyte-specific promoters can be used to guarantee the gene expression in these cells [208, 209]. Although the development of drug-delivery NPs that specifically target astrocytes is still limited, astrocytes present a rich repertoire of receptors, which may be used to specifically target drugs and NPs to them.

Aquaporin 4 (AQP4) is a water channel preferentially expressed on astrocytes and displays a wide range of functions, namely, regulation of potassium and calcium concentrations, osmotic pressure, waste clearance, neuroinflammation, and cell migration and synaptic plasticity [210, 211]. Interestingly, AQP4 is strongly expressed on the surface of astrocytes in the context of neurodegeneration [212]. Taking advantage of the preferential expression of this water channel on astrocytes, an anti-AQP4 antibody was conjugated with polymeric poly(glycidyl methacrylate) (PGMA) NPs to deliver the anti-oxidant resveratrol to tackle oxidative stress in the context of neurodegenerative diseases [110]. Resveratrol has shown poor bioavailability and rapid metabolization in vivo [213, 214]. Thus, the authors reported the accumulation of the AQP4-targeted NPs loaded with rhodamine B in GFAP-positive astrocytes, demonstrating the anti-AQP4 antibody targeting to astrocytes. AQP4-targeted NPs loaded with resveratrol were then administered in situ after optic nerve injury induction in adult female Piebald Viral Glaxo rats. The AQP4-targeted NPs were found to accumulate inside astrocytes and to effectively deliver resveratrol when administered to the site of injury. Furthermore, the targeted NPs were also able to rescue oxidative damage in the site of injury, as demonstrated by the reduction of immunoreactivity of 8-hydroxy-2’-deoxyguanosine (8OHdG) (a hallmark of oxidative damage in nuclear and mitochondrial DNA), as compared to non-targeted or non-loaded NPs [110]. Therefore, this study demonstrates the ability of the anti-AQP4 antibody to target NPs to astrocytes.

In another approach, the D4 monoclonal antibody that recognizes the GFAP protein preferentially expressed by astrocytes [215, 216], was linked to PEGylated liposomes [111]. The DiI fluorescent dye was integrated into the liposome’s bilayer allowing the visualization of the targeted NPs interaction with the astrocytes in vitro, through fluorescence microscopy. The specificity of the D4 antibody-conjugated liposomes to specifically interact with astrocytes was confirmed, since non-targeted or liposomes conjugated with a Control (non-specific) antibody were not visualized in the astrocytes. However, when administered to male Wistar rats by IV administration in the femoral vein, these NPs were unable to reach CNS astrocytes, mainly due to their inability to cross the BBB [111]. This work opens the avenue to speculate that these NPs may be useful in the context of diseases that present a weakened BBB or, furthermore, to functionalize these NPs with a second targeting ligand to allow their BBB crossing. In line with the former example, chitosan NPs functionalized with two commercially available antibodies, one targeting the transferrin receptor (widely expressed on BBB endothelial cells) and another targeting the bradykinin B2 receptor. Bradykinin B2 receptor (B2R) is associated with vasodilatation, neuroinflammation, and glucose uptake [217, 218]. B2R is not exclusive to astrocytes but is highly expressed in these cells [219, 220]. Therefore, an antibody anti-BR2, which is rapidly internalized after binding with a specific ligand, was employed in combination with transferrin in chitosan NPs to aid in overcoming the BBB [112]. These double-targeted chitosan NPs were tested in a BBB in vitro model to deliver siRNA to inhibit HIV-1 replication in astrocytes. SiRNA anti-SART3 and -hCycT1 genes, both important for HIV-1 replication in astrocytes, were employed. It was reported that the dual-targeted NPs penetrated across the human cerebral microvascular endothelial cells (hMCEC/D3) and accumulated in the human astrocytoma cells (U138-MG). This cell targeting resulted in a 6 times higher accumulation of siRNA in U138-MG cells, as compared to non-targeted NPs. Furthermore, the presence of the siRNA in these cells resulted in a gene knockdown of 81% and 67% for *SART3* and *hCycT1* mRNA, respectively [112].

Considering the small development of NPs specifically targeting astrocytes, it is of great interest to further explore more receptors that are exclusively or preferentially expressed by astrocytes in order to use them in NPs. For example, the N-acetylaspartylglutamate (NAAG) receptor, also known as metabotropic glutamate receptor 3 (mGluR3), is expressed in both neurons and astrocytes but their expression is enriched in astrocytes [221]. This receptor is activated by the neurotransmitter NAAG peptidase released by stimulated neurons [222] and its activation is believed to influence neuron and neurovascular stimulation in the context of schizophrenia and other neuropathies [222]. Moreover, a recent review highlighted the importance of some astrocyte receptors and transporters in the context of AD [223]. In particular, the excitatory aminoacid transporters EAAT1 and EAAT2, which although not exclusively expressed by astrocytes are in much larger amount in these cells [224]. In fact, EAAT are more active on astrocytes since they are responsible for 80% of the glutamate uptake [225]. Targeting these receptors would not only be promising to direct NPs to astrocytes but represents as well an opportunity to treat excitotoxicity in the context of neurodegenerative diseases [223, 226, 227]. Furthermore, the protein S100β is a calcium-binding protein abundantly expressed by mature astrocytes with the ability to be internalized [228, 229]. Thus, the coupling of targeting ligands for S100β to NPs may also present a capable strategy to target astrocytes for drug delivery. Finally, the active targeting of the cannabinoid receptors CB1 and CB2 present in glial cells, such as astrocytes and microglia, may help to control the neuroinflammation characteristic of several neurodegenerative diseases, by modulating the expression of inflammatory cytokines in these cells and their migration [230]. However interesting, NPs with targeting ligands that direct them to these receptors are yet to be explored.

### Targeting microglia

Despite being CNS resident immune cells, microglia do not develop from the neuroectoderm like other neural cells. They are derived from the yolk sac primitive macrophages and migrate to the CNS during embryonic development [231], representing 5 to 12% of all cells in the healthy CNS [232]. Physiologically, microglia have surveillance phenotype characterized by a ramified morphology and are the first line of defense against pathogens, promoting brain homeostasis and repair [233, 234]. Moreover, these cells have key functions in several processes such as neurogenesis, neural circuits refinement, and mediation of neurotransmission and synaptic pruning [235, 236]. However, in situations where the homeostasis in the brain is compromised, such as neurodegeneration or sustained inflammation, microglia changes their phenotype to an ameboid-like structure and alters their secretome, upregulating the expression of several cytokines, interleukins, and complement factors, enhancing and perpetuating neuroinflammation [232, 237]. Considering the characteristics of microglia as first responders to changes in brain homeostasis and their role in neuroinflammation, they appear as an interesting target for brain therapies. Indeed, several publications demonstrate a high internalization ability of activated microglia compared to non-activated [238–240]. Nonetheless, given the intrinsic phagocytic nature of microglia, concerns have been raised considering the specificity of microglial uptake of NPs, since NPs may just be recognized as pathogens [241, 242].

Microglia present a wide range of receptors, due to their surveillance function, so NPs can be tailored to take advantage of these receptors. Innate immune cells, such as microglia, have Pattern Recognition Receptors (PRRs) that have been used to target them [243]. These include Toll-Like Receptors (TLR), Receptors for Advanced Glycation Endproducts (RAGE), and Scavenger Receptors [244–247].

Choi and colleagues designed ceria-zirconia NPs (composed of Cerium and Zirconium) that specifically targeted microglia by conjugation with antibodies anti-CD11b (a receptor expressed on the surface of microglia and macrophages [248, 249]). In this work, the authors hypothesized that oxidative stress and inflammatory activation of microglia plays a role in neuropathic pain by sensitizing neurons, and tackled this by taking advantage of the anti-oxidant proprieties of ceria, particularly Ce^3+^ [250]. CD11b-targeted and non-targeted NPs labeled with FITC were incubated with microglia cells isolated from C57Bl/6 pups. Authors reported a higher percentage of FITC-positive cells with targeted NPs compared with non-targeted NPs, 80% and 40%, respectively. Regarding the induction of oxidative stress in microglia using *tert*-butyl hydroperoxide, a more pronounced reduction of ROS was observed when the CD11b-targeted NPs were added to the culture medium as compared to non-targeted NPs. Additionally, in cells pre-treated with lipoteichoic acid to induce the expression of iNOS, IL-6, and IL-1β (related to oxidative stress and inflammation) the treatment with CD11b-targeted NP, led to a 95%, 86%, and 91%, respectively, reduction in the mRNA levels of these genes. While the treatment with non-targeted NPs was only able to achieve reduction levels of 82%, 63%, and 71%, respectively. Moreover, CD11b-targeted and non-targeted NPs were administered using intrathecal injection in a neuropathic pain C57Bl/6 mouse model (spinal nerve transection). It was described a strong correlation between the FITC signal and the microglia-specific marker Iba-1, with co-localization observed in 84% of cells. Whereas, co-localization with the astrocyte marker GFAP and the neuron marker MAP2 was only detected in 26% and 11% of cells, respectively. Finally, the authors also observed a reduction in the hypersensitivity of these animals after treatment with CD11b-targeted NPs, compared with animals treated with non-targeted NPs [113], demonstrating the targeting ability of these NPs to microglia.

Despite these promising results, more targeting receptors and proteins specific to microglia are required to be explored in NPs development. For example, scavenger receptors are receptors present in cells of the immune system, having a wide range of functions, such as cargo transport inside the cell, lipid transport, recognition and removal of altered lipoproteins, and pathogen clearance [251]. Examples of scavenger receptors expressed by microglia are SR-A1 and CD36, which are used by microglia to bind and clear β-amyloid fibrils in the context of Alzheimer´s disease [252, 253]. However, these receptors are not fully specific of microglia since they are also expressed in macrophages, platelets, and endothelial cells. Therefore, careful consideration must be done when considering these receptors as targets for NPs targeting [254, 255].

Other interesting target is the transmembrane lectin sialic acid-binding immunoglobulin-like lectin H (Siglec-H) that in mice is able to discriminate microglia from CNS-bound macrophages and monocytes more accurately than CD11b or Iba-1 [256]. Further characterization (e.g. binding ligands and specificity, internalization mechanisms, etc.) and the discovery of a human homolog of this receptor may create the opportunity to design NPs to deliver therapies specifically to microglia [256]. Another receptor widely characterized and acknowledged to be microglia specific is the Cx3Cr1 receptor, also known as fractalkine receptor or G-protein coupled receptor 13 (GPR13) [257]. This receptor binds to the chemokine CX3CL1, also known as neurotactin or fractalkine. Moreover, the receptor P_2_ × _4_ is also an interesting potential target, since it is widely expressed in microglia and neurons but has a 3-fold increased expression in microglia under pathological conditions, such as neuroinflammation, hypoxia, and neuropathic pain [258, 259]. Although widely expressed in microglia, to this day there are no NPs developed to specifically target these receptors in these cells.

### Targeting oligodendrocytes

Oligodendrocytes are specialized cells of the CNS responsible for the myelination of neurons [260, 261]. The myelin sheath is a highly complex structure composed of 80% lipids and 20% proteins [261, 262] that provides insulating properties to neuronal axons which facilitate electrical signals transmission [261].

Given their unique characteristics, oligodendrocytes are among the most vulnerable cells in the CNS, and demyelination of axons is one of the hallmarks of neurodegeneration [260, 262]. As so, one potential therapeutic approach is to promote remyelination by inducing oligodendrocyte progenitor cells (OPC) to mature into oligodendrocytes and remyelinate the axons [263]. In order to promote remyelination by targeting OPC, Rittchen and colleagues developed PLGA NPs loaded with leukemia inhibitory factor (LIF), a robust pro-remyelination factor [114]. To achieve targeted delivery of the NPs to OPC, the authors used as targeting moiety antibodies anti-NG-2 chondroitin sulfate proteoglycan, a proteoglycan predominately expressed in OPC [264]. Three days after a 24 h treatment with PLGA-LIF NPs targeted to NG-2, rat OPC cultures presented a 33% increase in cells expressing myelin basic protein (MBP), a marker of mature oligodendrocytes, compared to non-targeted PLGA-LIF NPs. The remyelination potential of these NPs in vivo was tested in a mouse model of focal demyelinating lesion, in which the myelin toxin lysophosphatidylcholine (LPC) was administered to the corpus callosum by stereotaxic injection [114]. Eight days after the lesion, NG-2-targeted and non-targeted PLGA-LIF NPs were injected in the animals, and the effects were assessed 10- and 17-days post-administration. Using electron microscopy, a significant increase in the percentage of myelinated fibers per lesion and significantly thicker myelin sheaths were observed in animals treated with NG-2 targeted PLGA-LIF NPs compared to animals that received non-targeted NPs [114].

Interestingly, immunoglobulin M antibodies demonstrated the ability to target reactive oligodendrocytes and promote remyelination in a multiple sclerosis (MS) mouse model [265]. Inspired by this work, Tuerk and colleagues tried to identify DNA aptamers with the same binding affinity to myelin as the immunoglobulin M antibodies [266]. Authors identified a 40-nucleotide guanosine-rich DNA aptamer with anti-myelin proprieties when in a G-quadruplex structure (LJM-3064) [267]. In order to obtain the G-quadruplex structure, the biotinylated DNA aptamer was conjugated to a streptavidin core [268], resulting in a structure the authors called Myaptavin-3064 [267]. The capacity of this structure to promote remyelination in a mouse model of MS was demonstrated, but the specific interaction with oligodendrocytes was not tested [267]. In a recent work, the same group tested the affinity of Myaptavin-3064 to a human oligodendroglioma cell line (HOG) and mature oligodendrocytes differentiated from HOG cells [115]. Flow-cytometry data demonstrated that the binding of Myaptavin-3064 to HOG was increased upon differentiation with almost 90% of differentiated oligodendrocytes positive for Myaptavin-3064, while only 50% of HOG cells bound to Myaptavin-3064 with the same dose. The specificity of Myaptavin-3064 for oligodendrocytes was further confirmed with lung (L2) and kidney (BHK) cells, since flow-cytometry results indicated a residual affinity to these cells. Moreover, in primary cultures of adult rat cortical tissue, the authors identified that 97% of cells positive for the O4, an oligodendrocytes marker, were also positive for anti-streptavidin when co-cultured with Myaptavin-3064, while the co-staining was residual after culture with a control conjugate with a non-specific aptamer (LJM-3060) [115].

Another group linked the same aptamer (LJM-3064) to the surface of mouse mesenchymal stem cell-derived Exosomes to deliver cargo to oligodendrocytes [116]. In this work, LJM-3064 was employed not only as a targeting ligand for oligodendrocytes but also for the remyelinating capacity that it had demonstrated before as well [267]. The binding affinity of the exosome-aptamer conjugate (Exo-APT) was demonstrated in vitro in an oligodendrocytes cell line (OLN93). Exosomes, either targeted or untargeted with the aptamer, were then labeled with ATTO647N. Through flow cytometry analysis an increase in cell fluorescence was observed after incubation with Exo-APT compared to untargeted exosomes. Moreover, Exo-APT also promoted a significant increase in OLN93 proliferation compared to untargeted exosomes, assessed by BrdU cell proliferation assay [116]. Exo-APT or untargeted exosomes were administrated intravenously in mice before the induction of autoimmune encephalomyelitis (a mouse model commonly used to study MS [269]). A strong reduction in demyelination, a robust suppression in inflammation, and a reduction in the disease severity in animals administered with the Exo-APT were reported [116].

Taken together, despite promising, the work done so far to specifically target NPs to oligodendrocytes to treat brain diseases is still very scarce.

### Targeting neural stem cells

The loss of neurons is a major hallmark of neurodegenerative diseases; thus, an approach to tackle these diseases is the replacement of dead or impaired neurons. This can be achieved by stimulating neurogenesis, a process in which new mature neural cells are produced from neural stem cells (NSC) present in endogenous niches or engrafted by cell transplantation [270, 271]. The adult brain presents regions where NSC reside, the so-called neurogenic niches. The subgranular zone (SGZ) of the dentate gyrus and the subventricular zone (SVZ) of the lateral ventricles are two well-studied niches of NSC. The activity of these niches is crucial for neuroplasticity and learning. However, studies suggest that with aging a reduction of the proliferative, migratory, and integrative capacity of NSC takes place, which severely hampers neuroplasticity [271, 272]. Therefore, targeting endogenous NSC with drugs that promote their ability to proliferate, differentiate, migrate, and integrate may be advantageous to promote the replacement of the lost neural cells [273, 274]. However, NPs targeting the neurogenic niches and NSC is a field of research poorly explored and there is a demand to find targeting ligands that specifically direct drugs to NSC.

Schmidt and associates identified ligands by phage display technology with the ability to target neural progenitor cells (NPC) [118]. In this study, the ability of random peptides from a 7mer phage library commercially available to bind and be internalized by neurosphere cultures derived from the hippocampus of adult C57Bl/6 mice was evaluated. The authors tested 130 candidates for their binding efficiency for Nestin-positive cells in vitro. QTRFLLH and VPTQSSG peptides showed 10 to 20-fold increased binding to NPC compared with other peptides. Moreover, regarding cell specificity, QTRFLLH binding to NPC was significantly higher compared to Pan02 (pancreatic cancer cells), NIH3T3 (fibroblasts), H1299 (lung cancer cells), and HEK293 (human embryonic kidney cells). As for VPTQSSG, it exhibited lower binding affinity to NPC but higher cell specificity compared to QTRFLLH, with binding affinities 10 times lower to Pan02 and NIH3T3 and residual binding to H1299 and HEK293. QTRFLLH and VPTQSSG also revealed strong uptake by NPC. As adenoviruses present low infection efficiency of NPC [117], QTRFLLH and VPTQSSG were covalently linked to an adenoviral vector (wild-type capsid) expressing red fluorescent protein (RFP) to improve the viral delivery to NPC. Through immunofluorescence microscopy, it was observed the expression of RFP inside the NPC, supporting the hypothesis that these ligands can mediate adenovirus binding and uptake by NPC. Then, these viral vectors coding for RFP and linked with either peptide were injected into the hippocampus of a transgenic mouse model expressing GFP in Nestin-positive cells (pNestin-GFP) [275]. A strong specific co-localization of GFP and RFP was detected, suggesting that the peptides are efficient in guiding the adenovirus to Nestin-positive cells; whereas, the same adenovirus but linked to an unspecific peptide, led to almost no co-localization of RFP and GFP. The percentage of cells with RFP and GFP co-localization was 83.5% for the QTRFLLH peptide and 85.6% for the VPTQSSG peptide, whereas this percentage was 15.5% for the wild-type vector without any peptide and 8.6% for the adenovirus with the unspecific peptide [118]. Thus, these data indicate that these peptides mediate specific targeting to Nestin-expressing NPC.

The neurofilament light subunit (NFL) is known to present a strong interaction with NSC of the SVZ, showing a preferential accumulation in these cells in vivo after intra-lateral ventricular injections and the ability to induce their differentiation in vitro [276, 277]. Accordingly, it was demonstrated that the tubulin-binding site of the NFL (NFL-TBS.40–63), adsorbed to the surface of lipid nanocapsules (NFL-LNC), is able to guide lipid nanocapsules specifically to NSC in the SVZ [120].

Interestingly, besides being used to target the BBB, transferrin has as well been used to target NSC. In the work by Praca and colleagues, gold nanoparticles and gold nanorods were functionalized with medium density of transferrin peptides (between 169 and 230 transferrin peptides per NP) to direct the particles to the NSC [121]. Gold-NPs, with and without transferrin functionalization, were injected in the tail vein of adult (8 weeks old) C57Bl/6 mice. Irradiation with near-infrared light (NIR) was applied 1 h after administration to transiently open the BBB. Then, the animals were sacrificed 2 h after the NPs administration and their presence in the different brain regions was analyzed using mass spectrometry. As expected, the gold-NPs functionalized with transferrin preferentially accumulated in the brain compared to non-functionalized gold-NPs. Interestingly, after NIR irradiation, gold-NPs functionalized with transferrin were significantly accumulated in the SVZ (almost 0.2% of the injected dose). Without radiation, the percentage of these NPs accumulated in the SVZ was less than 0.1% of the injected dose and the NPs were more scattered in the brain and found preferentially in non-neurogenic areas. Gold-NPs without functionalization were found only residually in some non-neurogenic regions [121].

Altogether, these data demonstrate that although interesting, the targeting of NPs to NSC and NPC is still a very unexplored field.

### Limitations, successes, and strategies of brain-targeted NPs development for drug delivery

Despite the great potential of brain-targeted NPs to deliver therapeutic molecules to the brain, there is a need to better study the limitations and challenges of this strategy. In this regard, the concept of critical quality attributes (CQAs) has been established by the regulatory authorities to guide their development, characterization, and stability [278, 279]. The lipid composition of the NPs is a critical parameter to determine their proprieties and safety [280]. Therefore, the implementation of biocompatible and biodegradable materials [280–282] to develop safe NPs to be used in long-term therapies is key. The best composition of NPs is highly dependent on the intended use and, especially, on the cargo drug to be encapsulated [283, 284]. Physical characteristics, such as morphology, size, size distribution, surface-to-size ratio, and zeta potential are of the utmost importance to their safety and efficiency as delivery vehicles. Nonetheless, these characteristics are also highly dependent on their intended application, cargo drug, and composition. In general, the regulatory advice is that NPs should have a size lower to 100 nm [11]. In fact, smaller NPs are more easily eliminated by the kidneys, while larger NPs tend to be trapped in the lungs [282]. Regarding the size distribution, a polydispersity index (PDI) of 0.3 or below is considered adequate and reflective of a homogenous NPs population [285]. A large surface-to-size ratio (small size and a very large surface area) may lead to problems like limited drug loading, particle aggregation or friction, and a high clearance ratio [281]. The large surface area also increases their chemical reactivity, which may cause toxicity, namely through increased reactive oxygen species (ROS) production, neuroinflammation, and DNA damage [281, 286]. The zeta potential of NPs is determined by the presence of ionic lipids and/or charged surface ligands in their composition, which influences particle repulsion, aggregation tendency, and biodistribution [287]. Values between − 30 mV and + 30 mV are considered to keep stable particles suspension and enough inter-particle repulsion [278, 279]. Finally, the physical stability over time, namely particle fusion or aggregation, drug leakage, and chemical degradation of the lipids and their cargo, are also critical parameters that must be clearly evaluated several months post formulation [278, 279].

Alongside these issues, another limitation is the lack of specific targeting ligands to be used in NPs. In fact, although a preferential accumulation of the targeted NPs exists in the intended cells, some non-specific and potentially toxic accumulation in peripheral organs persists, especially in metabolizing organs such as the liver, lungs, and kidneys (Table 3). This may cause off-target adverse effects and hinder the therapeutic efficacy of the targeted therapies. The use of targets that are ubiquitously expressed through the body enhances this non-specific targeting. Ideally, a targeted drug-delivery approach for the brain using NPs should be able to overcome the BBB, specifically recognize the target cells in the brain aimed for treatment, enable endosomal escape after internalization, and release the cargo drug [288]. Thus, future research needs to focus on identifying more tissue and cell specific markers to be implemented in targeted therapies. An interesting approach to overcome this non-specificity may be the use of a double-targeting strategy. As seen in some reports targeting cancer cells [146, 162], the coupling of two different targeting-ligands to NPs can enhance their active targeting and improve their cargo delivery. Such dual targeting, where a targeting ligand is used to overcome the BBB and a second ligand to deliver the NPs to a specific cell population in the brain, can drastically improve therapeutic efficacy. Zhang and colleagues applied this strategy in the context of AD, using a peptide to target the BBB (TGN) and a second peptide (QSH) that binds to Aβ_1-42_ in poly(lactic acid) PLA NPs. Authors reported increased brain concentration and distribution in the Aβ_1-42_ plaques using the dual-targeted NPs [94]. These encouraging results, indicate that a dual targeting strategy to tackle brain diseases is a promising strategy. Nonetheless, whether it is the best therapeutic option or not it will have to be assessed on a case-by-case basis, as in some situations it will not be necessary, particularly given that some ligands may trigger this double targeting.

Furthermore, the production of targeted NPs with defined targeting, high quality and adequate for translational reproducibility remains a challenge. One of the key advantages of NPs is that their surface is highly tunable from a chemistry standpoint. In this regard the conjugation of the targeting ligand to the surface of the NPs becomes a critical aspect in the formulation [289, 290]. Two main strategies exist for the addition of targeting moieties to NPs, one-pot assembly and post-insertion through surface modifications [289]. In the one-pot assembly strategy, the targeting ligand is directly added to the lipid mixture prior to NPs formation. This is only feasible for targeting moieties able to endure exposure to organic solvents and high temperatures used during NPs production. Despite quite simple, this strategy presents a major hurdle, the orientation and density of the targeting ligand is completely unpredictable, resulting in a high percentage of ligand in the inside surface of the NPs. In the post-insertion strategy, the NPs are firstly formed and then a surface modification is performed by conjugation of the targeting ligand [289, 290]. This conjugation requires a chemical modification on the surface of the NPs by addition of functional groups that will react with reactive groups on the targeting ligands [290]. An important limitation of this strategy is the frequently observed low insertion yields of ligands in NPs [289]. Such low yields present a scalability problem since very high amounts of ligand are required to produce small amounts of targeted NPs, resulting in a very expensive manufacturing process.

Targeting ligand density in NPs defines much of the targeting abilities of the NPs since, if there is a too high ligand density, it may result in off-target binding in tissues with lower expression levels of the target receptor. On the other hand, if the density is too low, target cell uptake may be limited. Hence, targeting ligand density in NPs should match the receptor density in target cells [105]. Additionally, in vivo stability of the targeting ligands must also be addressed during NPs development [290–292]. In vivo, targeting ligands are subjected to a hostile environment promoted by degrading enzymes, pH, hypoxia, redox, and temperature variations. These are very important factors that might significantly impact targeted NPs therapeutic success [289].

To be successfully marketed, targeted NPs formulations need to have a large-scale manufacturing process. In addition to the issues related to ligand-NPs conjugation mentioned above, batch-to-batch variations in ligand density and stability, the choice of raw materials, synthesis processes, batch sizes, stability analyses, and documentation needs to be carefully considered [293].

The translation of a new therapy from a pre-clinical to a clinical investigation setting is always challenging [294]. For example, the most popular liposome production method is the lipid film hydration method, but the scaling up of this method from milliliters to liters batches, maintaining formulations with similar physicochemical proprieties is demanding [295]. Other methods like ethanol injection or reverse-phase evaporation are more easily adapted to an industrial setting; nevertheless, these methods face other challenges, such as optimization of particles size reduction, formulation homogeneity, and the removal of organic solvents and detergents [294, 295]. Attention must be drawn to several aspects in the early development stages to facilitate transition to an industrial setting. These include use of affordable and high grade raw materials; avoid low-yield and long synthetic reactions; avoid difficult to remove solvents and catalysts; use automation and closed circuit systems for improved safety, cost reduction, and evading errors; establish rigorous and adequate in-process and end-product quality controls; consider production risk assessment for hazardous batch contaminations and interference with the NPs formulation; use adequate methods for stability and shelf-life estimation; give special attention to formulations with a biological product, such as antibodies or proteins; and a cost-effective industrial production [293–295]. Overall, a multiparameter evaluation of the targeted NPs production process is needed to achieve successful scale-up manufacturing [293, 296], based in adequate in-process and final quality controls to ensure homogenous characteristics between batches and cost-effectiveness.

## Conclusions

Nanovesicles hold the promise to efficiently and precisely deliver diverse therapies into the brain to tackle neurodegenerative diseases. Nonetheless, these nanoparticles need to be specifically and efficiently delivered to the brain in order to potentiate their therapeutic outcomes without causing major side effects due to their accumulation in peripheral tissues. In this review, we summarized the different targeting ligands identified to deliver nanoparticles to specific cells in the brain. The research done so far in the development of brain-targeted NPs shows promising results in the targeted delivery and treatment of brain cells. In the future, this may result in high precision medicine, with reduced adverse side effects or unwanted therapy clearance from the body. However, much room for improvement still exists for these therapies to reach their full potential in the context of neurodegenerative diseases. For example, the identification of specific cell receptors expressed exclusively by each one of the different cell types would certainly prompt this field to the desired targeted drug delivery. Hence, we believe that it is important to keep focusing research endeavors on the screening of brain cell-specific receptors and in the design of high-affinity targeting ligands to be employed in the development of brain-targeted NPs carrying therapeutic molecules.

## Data Availability

No datasets were generated or analysed during the current study.

## Abbreviations

AD: Alzheimer’s disease
ALS: Amyotrophic Lateral Sclerosis
ApoE: apolipoprotein E
AQP4: Aquaporin 4
AZT: zidovudine
B2R: Bradykinin B2 receptor
BBB: blood-brain barrier
BDNF: brain-derived neurotrophic factor
CB: cannabinoid receptors
CNS: central nervous system
CPP: Cell-penetrating peptides
EAAT: excitatory aminoacid transporters
g7: glycopeptide 7
GAD: glutamic acid decarboxylase
GFP: green fluorescent protein
HB-EGF: Heparin-binding epidermal growth factor-like growth factor
HD: Huntington’s disease
HIV: human immunodeficiency virus
hMCEC/D3: immortalized human cerebral microvascular endothelial cell line
HOG: human oligodendroglioma cell line
i.p.: intraperitoneal
ICG: indocyanine green
ITZ: itraconazole
IV: intravenous administration
kFGF: kaposi fibroblast growth factor
LDL: low density lipoprotein
LDLR: Low-Density Lipoprotein Receptor
LIF: leukaemia inhibitory factor
LPC: lysophosphatidylcholine
MBP: myelin basic protein
Mel: Mellitin
mGluR3: metabotropic glutamate receptor 3
MJD: Machado-Joseph disease
MS: multiple sclerosis
N.A.: not applicable
N.T.: not tested
NAAG: N-acetylaspartylglutamate
nAChR: nicotinic acetylcholine receptor
NFL: neurofilament light subunit
NIR: near infrared
NPC: neural progenitor cells
NPs: nanoparticles
NPY: neuropeptide Y
NSC: neural stem cells
OLN93: oligodendrocytes cell line
OPC: oligodendrocyte progenitor cells
Pas: penetration accelerating sequence
PBCA: poly(n-butyl cyanoacrylate)
PCL: Polycaprolactone
PD: Parkinson’s disease
PEG: polyethylene glycol
PEI: polyethyleneimine
PGMA: poly(glycidyl methacrylate)
PLGA: poly(lactic-*co*-glycolic acid)
PRRs: Pattern Recognition Receptors
pVec: vascular endothelial-cadherin-derived peptide
QL: pentapeptide QLPVM
R8: arginine-rich peptide
RAGE: Receptors for Advanced Glycation Endproducts
RFP: red fluorescent protein
ROS: reactive oxygen species
RVG: rabies virus glycoprotein
SGZ: subgranular zone
Siglec-H: transmembrane lectin sialic acid-binding immunoglobulin-like lectin H
SVZ: subventricular zone
TAT: HIV-1 trans-activating protein
Tf: transferrin
TfR: Transferrin receptor
TLR: Toll-Like Receptors
TrkB: Tropomyosin receptor kinase B
U138-MG: human astrocytoma cell line
WHO: world health organization
